# Using an intermittent flow (“clamp and flash”) method to assess the readiness to wean from VA ECMO in adult and pediatric patients

**DOI:** 10.1051/ject/2025018

**Published:** 2025-09-15

**Authors:** James R. Neal, Pavel V. Mishin, Caitlin L. Blau, Devon O. Aganga, Troy G. Seelhammer

**Affiliations:** 1 Cardiovascular Surgery, Mayo Clinic 200 1st ST SW Rochester MN 55905 USA; 2 Critical Care and Anesthesia, Mayo Clinic 200 1st ST SW Rochester MN 55905 USA; 3 Pediatric Critical Care and Pediatric Anesthesia, Mayo Clinic 200 1st ST SW Rochester MN 55905 USA

**Keywords:** VA ECMO, ECMO Weaning, Heparin, Bivalirudin

## Abstract

*Background*: The use of VA extracorporeal membrane oxygenation (ECMO) for cardiac recovery is widely adopted, with extensive publications on assessing readiness to wean from VA ECMO. Techniques to reduce ECMO support vary, including reducing flows to a low continuous cardiac index, adding bridges, temporary flow cessation, or decreasing ECMO RPMs. *Method*: We propose an alternative method involving repeated cycles of 3–4 min of ECMO flow cessation (“clamp”) followed by a 30-second return (“flash”) of flow. This method requires additional anticoagulation to achieve an elevated ACT, targeting 220 s for adults and 210 s for pediatrics with heparin drip and bolus, or 240 s for adults and 225 s for pediatrics with bivalirudin drip and heparin bolus. During the clamp period, flow is stopped in adult ECMO circuits with a single venous line clamp, while in pediatric circuits, flow continues via the manifold shunt but is stopped in the arterial and venous lines with a single venous line clamp. Flashing the circuit resumes patient flow for 30 s to circulate stagnant blood. *Results*: This method significantly reduces support during the trial, which lasts one hour for adults and up to two hours for pediatric patients. The heart is unsupported 85–90% of the time, with an 85% decrease in cardiac support compared to low-flow trials. *Conclusion*: Since 2011, our center has used this technique without thrombotic complications when the protocol is followed. Most patients removed from ECMO did not require reinstitution, with rare cases needing VV support or VA support due to sepsis onset.

## Overview

Utilization of veno-arterial (VA) extracorporeal membrane oxygenation (ECMO) for support of a patient until heart function improves has long been reported in the literature [1, 2]. The timing and necessary recovery required to wean off ECMO have also been well described. Prior studies report the conditions needed for inotropes, heart function, and blood pressure, but often leave the act of reducing ECMO support short of a full explanation [1–3]. There have been studies reporting a temporary cessation of ECMO flow that appears to be in the range of minutes, followed by low flow with cardiac indexes ranging from 0.5 to 1 cardiac index (CI) or blood flow of 1–1.5 Liters per minute (LPM) in adult patients [2, 3]. Other studies have reported allowing the revolutions per minute (RPMs) of the centrifugal pump to be down-titrated to levels low enough to facilitate retrograde flow through the ECMO circuit, thereby generating a left-to-right shunt and subsequent diminishment of cardiac output by about 0.5–1 LPM [4–6]. While the operationalization may differ in each of these approaches, the ultimate goal of performing the turn down must be placed central to the discussion. In this context, the physiologic rationale is to diminish ECMO support to a threshold whereby ascertainment of tolerance of separation may be assessed. In some cases, such as a patient who has fully recovered cardiac function, prediction of successful separation may be straightforward. However, in many circumstances, a multitude of physiological perturbations challenge this assessment, which creates a clinical conundrum as to the potential tolerance of ECMO separation.

Staying on ECMO is not without risks, as previously published [7, 8]. The use of anticoagulants poses additional patient risk of bleeding while tempering the risk of thrombosis. Turning down the ECMO flow increases the risk of thrombosis due to the decreased velocity, leading to an increased length of time the blood stays in the circuit. Turning down the flow serves as a crucial step in ECMO weaning by making the heart produce more of the function of generating cardiac output. As the ECMO flow is titrated down, failure of the heart to tolerate the physiologic load, inclusive of both alterations in preload and afterload along with the requirement to generate additional native cardiac output may yield exacerbations in acidosis driven by increased lactate production, systemic hypotension, and/or disadvantageous changes in pulmonary vascular mechanics [2, 7]. The act of VA ECMO support also mechanically helps unload the right ventricle with continuous flow, regardless of how low the ECMO flow is titrated. It is important to recognize that any continuous forward flow will serve to temper the physiologic impacts of these changes while also providing an unloading function to varying degrees as defined by native reserve and amount of ongoing ECMO flow. Due to the multitude of variables contributing to this phase of care that complicate the determination of viability of separation, along with the potential risks associated with weaning as noted above, the timely, efficient, and effective separation is critical in reducing the morbidity and mortality profile of ECMO support.

## Description

From 2009 to 2011, our center had several ECMO patients who were expected to be successfully removed following weaning at a low continuous blood flow of about 0.75–1 LPM of blood flow with reasonable inotropic support, acceptable hemodynamic parameters, lactate levels, and arterial blood gases (ABG). Upon arrival in the operating room (OR) for decannulation, hypotension ensued during attempted decannulation, necessitating reinstitution of ECMO support. Right ventricular (RV) failure was postulated to be causative in many cases, which is similar to reported findings in other papers [9]. The need for reinsertion of cannulas is not without risk, given the difficulties associated with multiple re-cannulation of vessels, whether with a femoral or central approach [10].

These experiences yielded a reconceptualization of our approach to weaning and adaptation of a process similar to weaning from cardiopulmonary bypass. More specifically, this novel approach is contingent upon the intentional cessation of circuit flow while concurrently leaving cannulas indwelling and in continuity with the mechanical circuitry. To utilize this method, additional anticoagulation beyond standard levels on ECMO is required to prevent thrombus from forming in areas of relative or absolute blood stasis, including the cannulas. If managed like cardiopulmonary bypass (CPB), where heparin levels are maintained to produce an activated clotting time (ACT) over 480 s, it would be feasible for the ECMO circuit flow to be stopped for an extended period. This would be similar to CPB circuits being able to have flow cessation in them for over 1 h. However, this dramatic increase in anticoagulation would be fraught with bleeding risks. At our institution, a decision was made to perform a more “middle of the road” approach with a slight increase of the ACT allowing intermittent clamping (“clamp”) of the ECMO circuit between 3 and 4 min followed by a period of blood flow, which we call a “flash,” lasting for 30 s. By intermittently pausing and having periods of flow cessation, the unloading of the heart by the ECMO circuit is stopped, thereby unmasking physiologic reserve and hemodynamic tolerance of the patient.

The safe time of cessation was determined through consideration of the ACT. If it takes three and a half minutes (210 s) for an activated sample to thrombose, temporary cessation for that duration of time was deemed feasible without undue risk of circuit or cannula thrombosis. ACTs are drawn from the patient every 30 min during the trial, once a goal ACT is reached. Following this logic, the blood that had been stagnant is flushed back into the patient to allow new blood that had not been stagnant to come to rest in the circuit for another period of 3–4 min. By repeating this clamping and flashing, we could maintain circuit and cannula patency and protect the patient from either thrombosis or bleeding. This technique has now been utilized as a standard approach for the past 13 years. While we have no official number of patients to report, a conservative estimate is that many hundreds of adult-sized patients, from teenagers to eighty-year-olds and over 65 neonatal and smaller pediatric patients, have had this trial implemented during their ECMO course. Importantly, there have been no occurrences of loss of circuit integrity or patient thrombosis during these clamp flash trials when the protocols have been followed.

During the 30-second flash period, the blood flow of the ECMO circuit is set so that the circuit volume that was stagnant is completely flushed into the patient. At our center, the adult Cardiohelp (Maquet Rasatatt, Germany) is utilized with a circuit prime volume of approximately 600 mL (including cannulas). Thus, the circuit flow during the flash is set to 1.2 LPM for 30 s. The RPMs that provide the patient with 1.2 LPM of flow during the flash period are maintained during the clamp period. Our adult circuit does not contain shunts, bridges, or manifolds. While clamped, there is complete cessation of blood flow in the circuit. In addition, during the clamp period, the sweep is shut off to prevent the blood in the oxygenator from becoming hypocapnic and hyperoxemic. Only just before and just after the flash, is there a sweep going through the circuit. The sweep is adjusted to maintain appropriate pO_2_ and pCO2 levels coming out of the oxygenator during the flash. By having these intermittent periods of flow over an hour, the patients’ RV is unsupported by ECMO for the large majority of the time (Table 1).

**Table 1:** Comparison of cardiac index (CI) for a one-hour clamp flash trial with a 3.5-minute clamping period versus a continuous low flow trial for a hypothetical adult and pediatric patient. Note: Percent decrease in total support – L/min and time is a comparison between continuous low flow and clamp flash trials.

	Weight (kg)	BSA (m^2^)	Trial type	CI during flow (L/mim/m^2^)	Flow rate (L/min)	Total support (L/h)	% Decrease in total support (L/h)	Supported time (min)	% Decrease in support minutes (L/h)
Adult	80	2	Continuous low flow	0.5	1	60	–	60	–
	80	2	Clamp flash	0.3	0.6	9	85	7.5	88
Pediatric	4	0.48	Continuous low flow	0.25	0.12	7.2	–	60	–
	4	0.48	Clamp flash	0.29	0.14	1	86	7.5	88

With the use of the clamp flash trial in our pediatric patients, several modifications are required. Our primary ECMO circuits have consisted of, depending on patient size, either a Pedimag or Centrimag pump head (Abbott, Abbott Park, USA) and either a pediatric QuadroxiD (Maquet, Rastatt, Germany) or, more recently, an AMG PMP oxygenator (EuroSet, Medolla, Italy). Our center also includes a recirculating manifold that is either 1/8” or 3/16” diameter tubing with a flow between 0.12 and 0.6 LPM. The overall circuit volume is approximately 230 ml, including the arterial and venous lines of approximately 60 ml each. In larger pediatric patients, our center employs a Cardiohelp 5.0 (Maquet Rasatatt, Germany) with a modified 1/4 or 3/8 inch venous line and a modified 1/4 arterial line with a 3/16 inch recirculating manifold. In our pediatric population, given that we have a manifold, flow is maintained in the circuit, pump, and oxygenator during the clamp periods, but not in the arterial and venous lines to the patient. During our flash periods, our center aims to only have enough flow to the patient to change over the 60 mL present in each of the arterial and venous lines. The patient flow during the flash is set between 0.12 and 0.14 LPM for 30 s. The venous line is reclamped after the flash period. During the clamp period, the tubing clamp is placed only on the patient side of the venous line before the manifold re-entry. Similar to adult patients, sweep is only provided to the oxygenator during the period from just before until just after the flash. This prevents the circuit from becoming hypocapnic and hyperoxemic. Careful control of the sweep and fractional delivered oxygen (fdO2) is performed by the perfusionist or ECMO specialist. If needed, a circuit arterial blood gas (ABG) is performed during the clamp period to assess the gas settings during the flash. For both adult and pediatric patients, ABGs and lactates are drawn every 30 minutes during the trials.

Pediatric patients can have a more varied heart physiology than adults [11]. Children with congenital cyanotic heart lesions need special attention in controlling the paO2 and paCO2. During the flash, the goal is not only to prevent excessive hemodynamic support but also to prevent excessive respiratory support. Given this, after the second or third clamp, a blood gas off the manifold line is used to assess the circuit’s paO2 and paCO2, which is provided during the flash. For patients with cyanotic defects, the goal is not to elevate the SaO2 more than 2 to 3 mmHg above the patient’s baseline during the period of clamping. This prevents the flash from over-supporting the patient and misrepresenting the child’s respiratory ability during the clamp periods.

**Figure 1:**
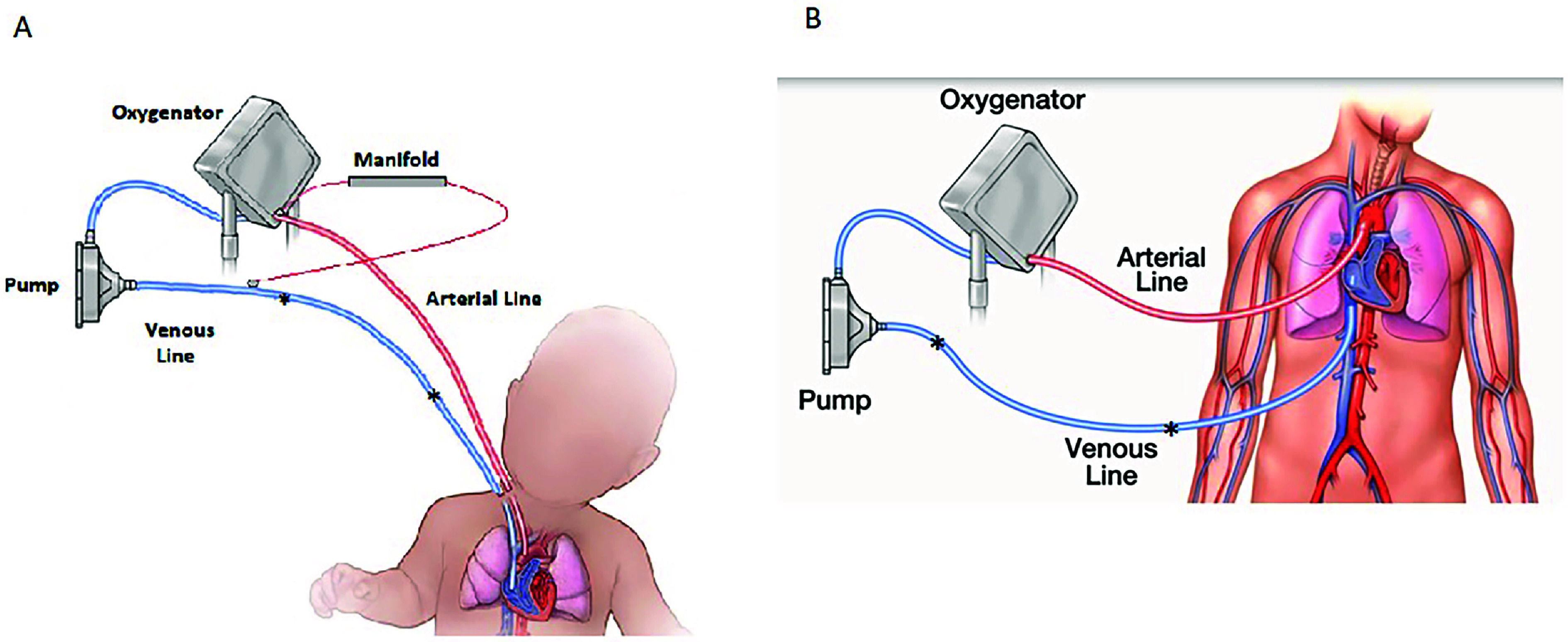
A. Representation of the pediatric circuit with recirculation manifold and patient. The tubing area between the two * represents the area where intermittent clamping would occur in varied locations. B. Representation of the adult ECMO circuit and patient. The tubing area between the two * represents the area where intermittent clamping would occur in varied locations.

An additional consideration during the trial is the location of the clamp placement on the venous line during flow cessation. The area that was clamped is varied during each subsequent clamping to prevent excessive wear and/or future thrombin/thrombus buildup at that location of the clamping (Fig. 1). This can sometimes happen after an unsuccessful wean. While minor and insignificant on the venous side of the circuit, this is a concern on the arterial limb due to the potential embolic risk to the patient’s systemic arterial side. Before implementing the purposeful relocation of the clamping location, this buildup of thrombotic deposition was noted on the venous line, which has been dramatically reduced following integration of this relatively minor, but important, protocol adjustment. The arterial line should be free of any red cell incorporated thrombus laydown and assessed for this before starting a clamp/flash trial, as this thrombus laydown could become mobile during the stagnant period. Then, during the flash, be sent into the arterial cannula or the patient.

The use of heparin as the only means of anticoagulation for ECMO has dramatically decreased over the last 14 years. Our center, in addition to many others, has increased the use of direct thrombin inhibitors (DTIs) to offset the need for anti-thrombin supplementation [10, 12]. Heparin is advantageous when protecting blood in states of stasis and from thrombosis, as compared to using DTIs only. Additionally, an issue with using DTIs is that there is a prolongation of the ACT that is artificial [13, 14]. Normally, this is not a concern since APTTs are used more often in ECMO. However, during a clamp and flash trial, the ACT is the critical lab value due to its quick result. This can lead to a situation where using artificially elevated ACT levels, whose absolute value was thought to be safe, is inadequate, yielding an elevated and difficult to quantify risk for thrombosis. Given the inaccuracy of the ACT in the context of concurrent bivalirudin administration, a solution was deemed necessary. Our center looked at previous research showing a direct, limited correlation between ACT and APTT values with heparin [15]. The study showed that low-level heparin administration is reflected in the values of these two tests. Consequently, our protocol was modified to apply these findings and compare the APTT and ACT values with a bivalirudin drip only versus the values on ECMO patients with a heparin drip. The difference in ACT values at similar APTTs led us to believe that the bivalirudin skews the ACT approximately 20–30 s higher. Normally, adult patients on a therapeutic bivalirudin infusion should have an ACT of roughly 230–250 s to start the clamp flash trial after a heparin bolus. In the pediatric setting with patients on a bivalirudin drip, the goal ACT after the heparin bolus is between 210 and 225 s, since only the arterial and venous lines are left stagnant during the clamp period (Table 2). Previously published institutional experience with bivalirudin, as well as numerous meta-analyses of retrospective studies, supported the viability and potential superiority of bivalirudin as compared to heparin-based systemic anticoagulation in ECMO [16]. Despite this reassuring data, it is essential to recall important limitations of bivalirudin therapy. Of these, the most important with regard to the application of clamps on ECMO circuitry is the organ-independent proteolysis of the parent compound, resulting in loss of regional anticoagulation in areas of relative or absolute blood stagnation. In effect, in areas of stasis, the drug is metabolized without requiring circulation through an effector organ, and as such, fresh delivery of bivalirudin is required to maintain systemic anticoagulation. Due to this concern, during clamp flash trials, heparin boluses are used concurrently with bivalirudin drips to ensure the appropriate presence of the desired intensity of systemic anticoagulation within the areas of stagnant blood flow. Another important note when performing these trials is the timing of ABGs. Our center finds the easiest time to draw an ABG from a patient is just before flashing for the 30-second period. The lab technician will inform the perfusionist/ECMO specialist once completed with the draw from the arterial line, so that flashing can commence. If there are concerns with the patient’s respiratory status, these values will mostly reflect how the patient can tolerate being off ECMO ventilatory support.

**Table 2:** Comparison of activated clotting times (ACT) targets during a clamp and flash trial with heparin only versus a heparin bolus and bivalirudin drip. I-stat ACT machine with kaolin cartridges was used for these target ACTs.

	ACT target with heparin (s)	ACT target with heparin + bivalirudin (s)
Adult	210–220	230–250
Pediatric	200–215	210–225

## Discussion

Numerous approaches to the weaning of VA ECMO have been described, including in the terminal phases. However, our clamp flash trial can serve as an alternative to these approaches that hybridizes the ready availability of restoration of flow with the ability to assess physiologic reserve in the absence of ongoing ECMO support. This is an essential component to the holistic comprehensive assessment because it facilitates determining physiologic reserve in the context of zero mechanical circuit flow while concurrently enabling the rapid re-application of support as necessary. Further, it facilitates a variable duration of assessment not confined to conventional trial-offs. This latter piece is a potent advantage of this approach because it enables an individualized method that recognizes the variation inherent in patient presentations. In our opinion, the clamp flash trial offers superior flexibility in duration of the terminal weaning trial in a manner that mitigates thrombotic risks and protects against undue, and potentially deleterious, physiologic strain. In assessing a patient’s readiness for ECMO decannulation, looking at the patient’s ejection fraction, CI, inotropic support, lactate levels, systolic blood pressure, and blood gases are all assessed during the clamp flash trial.

The clamp intervals must be thoroughly timed without guessing or lapses of flashes following stagnation periods. Improper timing could lead to thrombosis of the circuit, given the ACTs during this trial. Our center allows personal use of either a watch timer or a phone timer and does not use a wall clock as a timer. In our institution, the thought of increasing the anticoagulation further to increase the length of clamping to over 4 min was deemed not to be worth the increased risk of bleeding. When utilizing a 4-minute clamping interval for an hour trial, the patient is unsupported for 90% of the trial time. This has provided adequate information to assess whether the heart is ready to be weaned and discontinued from ECMO support. In some isolated patients (fewer than 8) that were weaned, ECMO had to be reinstituted after 24 h due to issues that were unforeseen during the initial weaning phase. The majority of these isolated patients who required reinstitution were due to a secondary insult unrelated to the initial indication, such as new onset septicemia or respiratory failure.

In the pediatric setting, previous studies have reported leaving cannulas connected to the patient with heparin flushes to keep them patent. This would prevent the need for re-cannulation if ECMO reinstitution was necessary [17, 18]. Other centers have reported using a bridge next to the cannulas to keep circuit flow high and patient flow low [19, 20]. Another center reported a transition from VA to VAV as a weaning method [21]. Our clamp flash trial can serve as an alternative to these approaches.

Once a clamp flash trial has been completed, and if decannulation is not imminent, the previously administered exogenous unfractionated heparin will wear off between 1 and 2 h, with minimal concern for bleeding. Inherently, there are some contraindications for using heparin, such as exacerbation of existing intracranial hemorrhage or in patients with known or suspected type 2 heparin-induced thrombocytopenia thrombosis (HITT). Fortunately, these patients are a minority in our overall ECMO patient population. Therefore, this trial has been used on the great majority of our adult and pediatric VA ECMO patients with noteworthy success. In the occurrences where there would be a contraindication to heparin, the broadly described and more conventional low-flow techniques continue to be utilized. Despite the recent interest in the retrograde flow techniques as reported in the literature, our center has not yet trialed this approach or compared it to the clamp-flash trial technique. Further, our extensive use of the Cardiohelp System on adult patients and some large pediatric patients precludes this approach due to embedded safety features integrated into that device that prevent retrograde flow [22]. While certainly a unique technique, we feel that many of our adult patients would fail weaning with a reversed flow, a left-to-right shunt of between 0.5 and 1 LPM, as reported in other papers.

While these trials give great data on how a patient will wean, there is a need for an increased level of staffing during the clamp and flash period. Either a perfusionist or an ECMO specialist needs to be available to perform the clamping/unclamping and timing of these events, along with the manipulation of the sweep and FDO_2_. The ability to perform these tests in the setting of an intensive care unit (ICU) is a valuable and effective way to prevent a risky and unnecessary OR trip, especially if a patient is not separable from ECMO. This also avoids costs from being accrued for the OR time, personnel, and related resources if the patient were unable to pass a clamp flash trial test.

The ACT machine that we used at our center is the i-STAT (Abott POC, Princeton, NJ) with kaolin cartridges. As reported in previous papers, there are some differences in ACT machines. Our values for a goal ACT may differ from other institutions, depending on their ACT machine or activator [23]. Given this, ECMO centers wishing to use our technique will need to digest this information and evaluate what their target ACTs should be before implementing a clamp flash trial.

The physiologic rationale and positive single-center experience over the past 13 years has yielded a highly favorable impression of the clamp flash trial at our center in assessing for ECMO removal readiness in a way that balances the risk of bleeding and thrombosis in a relatively short period. The potential benefits of this method are striking about the resilience of artificial support through ECMO flow cessation, with the patient being unsupported for 85–90% of the duration of the trial, which itself may be safely extended to facilitate the holistic ascertainment of physiologic reserve in the absence of circuit flow. Additionally, during the trial, support flow is reduced by 90% from the ECMO circuit as compared to a low continuous flow state. This approach compares favorably to a low continuous flow trial with no unsupported periods or a single time-limited trial achieved through the isolated application of a clamp to terminate flow. Our center’s experience with the clamp flash trial is defined as a reliable, effective, and efficient method to assess the readiness of our patients to be weaned from VA ECMO.

## Limitations

Future studies into this technique, as compared to other weaning techniques, would be the next logical step. The clamp flash technique, at this time, is being reported at a single site and lacks a scientific comparator, and has limited data on certain subgroups. While using the technique for over 13 years, the data from the trials were not always recorded or documented fully in the patient record. While reporting of data points from patients would give credibility to this technique, we do not have complete results to be able to pull from. Given this, we present this paper as a technical article with patient estimates. Our center will look to future data publication with improved recording of these trials in the future. We stress that utilizing the technique is safe when following our paper’s outlined instructions.

## Data Availability

No data were analyzed in this paper. Given this, we do not have a repository of data for supplementary material to acknowledge.
